# Toward ambulatory monitoring of vocal behavior at the physiological level using deep ensembles and Bayesian neural networks

**DOI:** 10.1121/10.0039842

**Published:** 2025-11-11

**Authors:** Zhaoyan Zhang

**Affiliations:** Department of Head and Neck Surgery, University of California, Los Angeles, Los Angeles, California 90095-1794, USA

## Abstract

Currently, diagnosis of voice disorders is often made when patients visit the clinic, by which time speakers already experience vocal difficulties. The goal of this study was to develop a voice inversion system that predicts how speakers modulate vocal physiology from the produced voice, toward early detection of unhealthy vocal behavior. Two neural networks, a Bayesian neural network and a deep ensemble of neural networks, were developed that predict changes in vocal physiological parameters and their confidence intervals. Comparison to human data showed that both networks were able to predict meaningful differences in vocal behavior across subjects, demonstrating their potential toward ambulatory monitoring of vocal behavior at the physiological level.

## 1. Introduction

Voice disorders have been estimated to affect approximately 30% of the adult population in the United States at some point in their lives, with about 7% of individuals affected at any given point in time.^1,2^ Due to limited access to the larynx, diagnosis is often made when patients visit the clinic, at which time the voice disorder often has already progressed to a point that speakers experience vocal difficulties or noticeable voice changes have occurred. Early diagnosis outside the clinic is difficult, as it is not easy to see into the throat without specialized equipment. However, unhealthy vocal behavior (e.g., tendency to talk loud or tightly squeeze the larynx), if not identified and intervened in a timely manner, may lead to repeated vocal fold injury and more permanent voice disorders.

Currently, there are no validated voice monitoring systems that allow speakers, particularly voice professionals such as teachers and singers, to monitor their vocal health and identify unhealthy, abusive vocal behavior outside the clinic. While there have been much research efforts toward ambulatory monitoring of the voice outside the clinic,^3–5^ they often focus on monitoring the produced voice outcomes (e.g., pitch, loudness, and voice quality) rather than how speakers modulate their vocal physiology as they speak (e.g., how much lung pressure is used and how strongly they adduct or stiffen the vocal folds). While changes in the produced voice outcomes provide important insights into potential pathophysiology, inferring the underlying changes in vocal physiology from the produced voice outcomes (the inverse problem) is not always straightforward. This makes it difficult to identify unhealthy, abusive vocal behavior from the produced voice alone, particularly in an ambulatory setting outside the clinic when endoscopy is often unavailable.

A main challenge in solving the inverse problem is the lack of experimental data on how speakers modulate vocal fold geometry and mechanical properties during phonation, due to limited access to the larynx, which makes it difficult to relate such physiological parameters to the produced voice outcomes. As a result, previous studies often have to rely on a computational voice production model to establish the relationship between vocal physiology and the produced voice outcomes, which is then used to solve the inverse problem (e.g., Refs. 6–13). More recently, a simulation-based machine learning approach was proposed^8–13^ that uses data generated from computational voice simulations to train neural networks to map the produced voice outcomes to the underlying physiological parameters. When data are generated using a three-dimensional continuum vocal fold model, such neural networks can estimate realistic, directly measurable, and clinically relevant physiological parameters, including vocal fold geometry, stiffness, glottal gap, and subglottal pressure.^9^ In our previous studies, validation in excised larynx experiment^9^ and in a single-subject study^10^ showed that the neural network was able to predict subglottal pressure with reasonable accuracy. Comparison to magnetic resonance imaging showed that the network also estimated vocal fold geometry with reasonable accuracy.^9^ A similar simulation-based machine learning approach using lumped-element vocal fold models^8,11–13^ was also reported to show similar performance in predicting the subglottal pressure.

A limitation of these recent studies is that the neural networks provide only point estimate predictions without any information on the uncertainty of the predictions. Uncertainty information is essential to the interpretation of the predictions and the clinical decision-making process. One way to obtain such information is to train neural networks in the Bayesian framework (e.g., Refs. 14 and 15), in which the weights and biases of the neural networks are considered as distributions rather than a single value, as recently implemented for voice inversion in Ref. 13. These distributions are then estimated from the training data. Alternatively, a distribution of each weight and bias can be estimated from an ensemble of neural networks that are initialized randomly and trained independently.^16,17^

In this study, we report our recent effort in developing neural networks for voice production inversion that not only predict vocal physiology but also provide a confidence interval (CI) of the predictions. Two approaches were considered, including a Bayesian neural network (BNN) and an ensemble of neural networks. The neural networks were trained using the same simulation data generated from the three-dimensional model in Refs. 9 and 10, which allow estimation of clinically relevant parameters. The potential of the neural networks toward ambulatory monitoring of vocal function and health was then evaluated by applying to data collected from three human subjects. We particularly focused on the neural networks' capabilities in predicting subglottal pressure and vocal fold adduction—two important factors contributing to risk of vocal fold injury and vocal health.^18,19^

## 2. Method

### 2.1 Training data

Data generated from voice production simulations using a three-dimensional computational model of voice production^9,20^ were used for neural network training in this study. The simulations involved parametric variations in the subglottal pressure and vocal fold geometry and stiffness, a total of nine control parameters (Table 1, output parameters), as described in Ref. 9. The ranges of control parameter variations were determined based on values reported in the literature, and encompass typical conditions of voice production in males, females, and children, as well as voices of varying qualities. A total of 2 21 400 simulations were performed. For each condition, voice features were extracted from the produced voice outcomes. These include the fundamental frequency (*F*0), sound pressure level (SPL), cepstral peak prominence (CPP), harmonic-to-noise ratio (HNR), subharmonic-to-harmonic ratio (SHR), the differences in amplitude between the first harmonic and the second harmonic (H1-H2), the fourth harmonic (H1-H4), the harmonic nearest 2 kHz (H1-H2k), and the harmonic nearest 5 kHz (H1-H5k) in the spectrum of the time derivative of the glottal flow waveform, mean (Qmean) and peak-to-peak amplitude (Qamp) of the glottal flow waveform, closed quotient (CQ) of the glottal flow waveform, maximum flow declination rate (MFDR), and maximum flow acceleration rate (MFAR).

**Table 1.** Input and output of the neural networks.

Input (voice features)	Output (physiological parameters)
*F*0, SPL, CPP, HNR, SHR, H1-H2, H1-H4, H1-H2k, H1-H5k, Qmean, Qamp, CQ, MFDR, MFAR	Vocal fold (VF) length (front-back; mm), VF vertical thickness (mm), VF body-layer depth (left-right; mm), VF cover-layer depth (left-right; mm), VF body-layer longitudinal stiffness (kPa), VF cover-layer longitudinal stiffness (kPa), VF transverse stiffness (kPa), glottal angle (VF approximation; degree), subglottal pressure (Pa)

In this study, neural networks were trained to estimate the nine physiological control parameters (Table 1, neural network output) from the 14 voice features (Table 1, input to the neural network). All data were *z*-scored before training. Similar to Ref. 9, this study intentionally did not include any features characterizing vocal fold vibration so that application of the trained networks does not require specialized equipment (e.g., endoscopy) that may not be readily available outside the clinic.

### 2.2 Neural network training

Each neural network included an input layer of voice features, four hidden layers with 200 neurons each, and an output layer. This configuration was selected based on previous studies using different number of layers and neurons.^9,10^ Two approaches were used to train a neural network that provides both predictions and their uncertainty intervals: deep ensembles and BNN. The neural network training was implemented in matlab Deep Learning Toolbox (MathWorks, Natick, MA, version 2019 b).

In the first approach, an ensemble of 100 neural networks were trained independently using the simulation data, similar to the procedure described in Ref. 16. For each neural network, the weights and biases were randomly initialized at the beginning of the training, and the training data were also randomly divided into sets of training (70%), validation (15%), and testing (15%). Each neural network was trained to minimize the mean squared error with regularization between the truth and network prediction, using the scaled conjugate gradient method. For a given input vector of voice features, these 100 neural networks provide a distribution of predictions, from which the mean value and a 95% CI are calculated.

Unlike the ensemble of neural networks with a single deterministic value for each of the weights and biases, BNNs model each weight and bias as a distribution.^14,15^ In this study, a BNN was trained with the posterior distributions of the weights and biases approximated with multivariate Gaussian distributions. The mean and standard deviation of these distributions were estimated by minimizing the evidence lower bound using the ADAM optimizer. For prediction, the posterior distributions were sampled 100 times for each input. This results in 100 estimates of the physiological control parameters, from which the mean value and a 95% CI are calculated.

### 2.3 Performance evaluation using human data

The performance of the neural networks was evaluated against human data. Acoustic and aerodynamic data were collected in human subjects producing utterances of five repetitions of the syllable /pa/ at different loudness levels. This speech task was chosen because the intraoral pressure during the /p/ is often used as an indirect estimate of the subglottal pressure during the following vowel /a/, which allows us to quantitatively evaluate the network performance in predicting the subglottal pressure. In addition, consecutive consonant–vowel sequences as the /pa/ require alternating vocal fold adduction and abduction, which allows qualitative evaluation of the prediction accuracy of the glottal angle. The results below are based on data from three subjects, including two males (ages 45 and 15 years) and one female (age 60 years).

The produced speech sound was measured using a $\frac{1}{2}$ in. B&K microphone. The oral volume flow rate was measured using a pneumotachograph attached to a circumferentially vented facemask placed against the speaker's face. The intraoral air pressure behind the lips was measured using a pressure transducer connected to a catheter, which was passed through a fitting in the facemask and was held between the lips into the oral cavity. Speakers were instructed to produce the utterance at varying loudness levels, ranging from soft, comfortable, to loud, without prescribed pitch or loudness levels.

From the recorded sound pressure data, SPL at 30 cm from the lips was calculated, and *F*0, CPP, HNR, SHR, and SPL were extracted using the software VOICESAUCE.^21^ Because the neural network was trained using simulation data produced without a vocal tract, the measured SPL was subtracted by 15 dB to correct for the effect of vocal tract resonance, similar to Ref. 9. This correction factor was determined based on SPL differences between conditions with an /ɑ/ vocal tract and without a vocal tract.^9^

Since the glottal flow is almost impossible to measure in humans, the oral volume flow rate was inverse filtered to obtain the glottal flow waveform using the INVF software,^22^ from which the glottal flow-related measures (Qmean, Qamp, CQ, MFDR, and MADR) and spectral shape measures (H1-H2, H1-H4, H1-H2k, H1-H5k) were extracted.

The peak intraoral pressure during the plosives was identified for each /p/ segment. Linear interpolation between the peak intraoral pressures of two consecutive /p/s was used to approximate the subglottal pressure during the vowel /a/ in between the two /p/'s, which was used as the ground truth subglottal pressure in this study to evaluate the network performance in predicting the subglottal pressure.

For the subglottal pressure, for which experimental data were available for comparison, the performance of the neural networks was evaluated by the mean absolute errors (MAE) and the mean absolute percentage errors (MAPE) between the prediction and the experimentally measured subglottal pressure. The performance of the CI was evaluated by the prediction interval coverage (PIC) or the percentage of times the experimentally measured subglottal pressure falls within the predicted 95% CI. For the glottal angle, for which no experimental data were available, the prediction performance was only qualitatively evaluated against expected vocal fold adduction/abduction behavior during plosive-vowel transitions.

## 3. Results

### 3.1 Comparison between neural network estimates and experiment

**Fig. 1.**
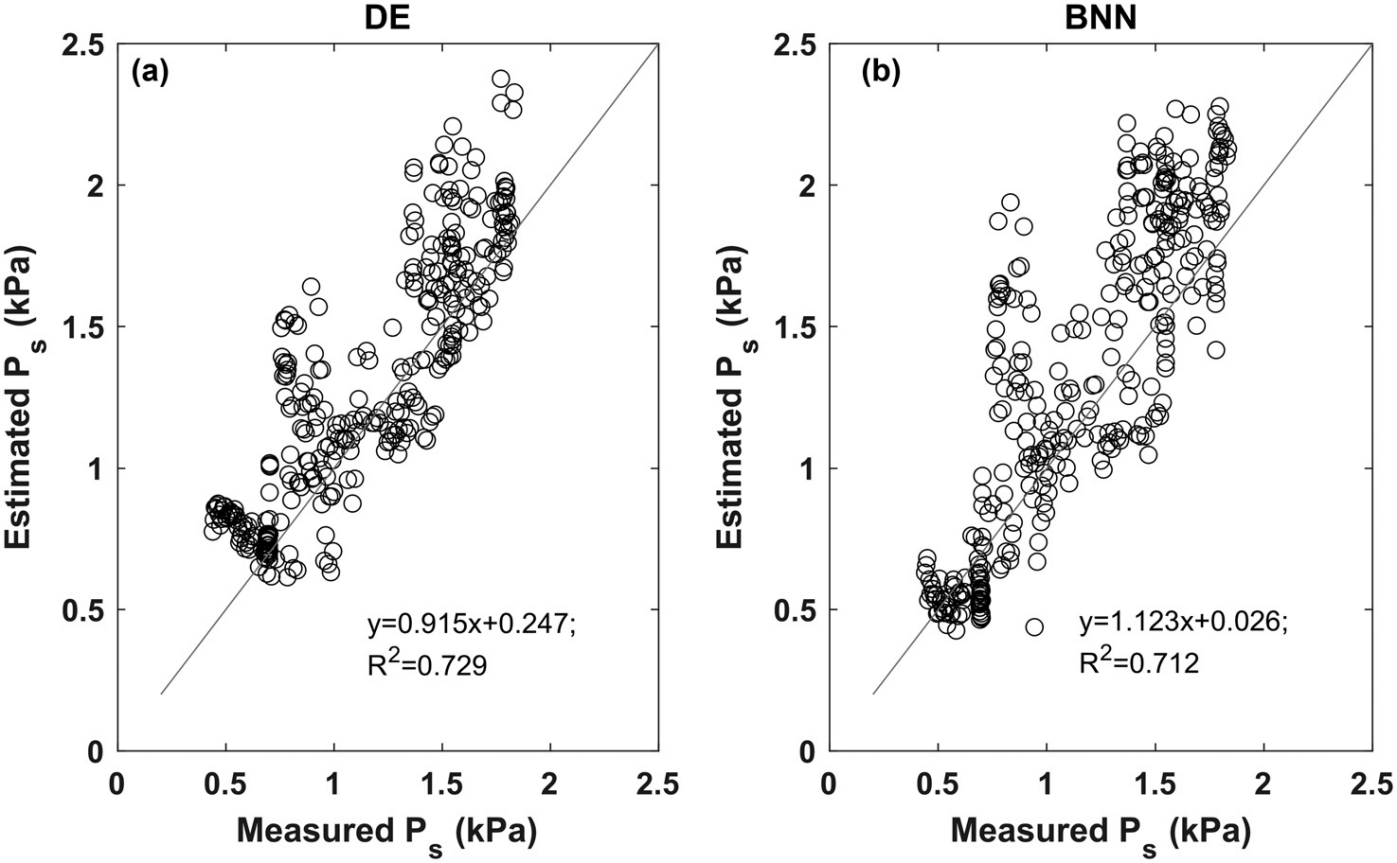
Deep ensemble (DE) and Bayesian neural network (BNN) estimated subglottal pressure (P_s_) as a function of experimental measurement. The solid line is a line through the origin with a slope of 1.

Figure 1 compares the subglottal pressure estimated from the two approaches [deep ensemble (DE) of neural networks and the BNN] and those measured experimentally, for data from all three subjects. In general, the estimates from both approaches followed the experimental values. The neural networks tended to slightly overestimate the subglottal pressure, with a linear regression slope of 0.915 and an *R*^2^ value of 0.729 for the DE and a linear regression slop of 1.123 and an *R*^2^ value of 0.712 for the BNN. For the DE, the MAE was 212 Pa (Table 2), which is slightly better than previous studies involving human subjects^10,11^ but larger than that in previous studies using excised larynges.^8,9,12^ The MAPE was 22.4%, comparable to other studies^8,10,11^ but larger than Ref. 12.

**Table 2.** Point estimate accuracy and confidence interval (CI) for the subglottal pressure and glottal angle for the three subjects (M1, M2, and F1). The CI values include both the mean and standard deviation of the CI across the utterances. MAE, mean absolute error; MAPE, mean absolute percentage error; PIC, prediction interval coverage. MAE, MAPE, and PIC are not available for the glottal angle due to the lack of experimental data.

		Deep ensembles	Bayesian neural network
Subglottal pressure (kPa)	MAE	0.212	0.264
	MAPE	22.4%	24.4%
	PIC	99.6%	99.6%
	CI all subjects	0.528 ± 0.125	0.743 ± 0.053
	CI (M1)	0.518 ± 0.089	0.757 ± 0.056
	CI (M2)	0.600 ± 0.161	0.757 ± 0.046
	CI (F1)	0.479 ± 0.071	0.726 ± 0.051
Glottal angle (°)	CI all subjects	1.592 ± 0.352	1.581 ± 0.126
	CI (M1)	1.608 ± 0.305	1.599 ± 0.122
	CI (M2)	1.593 ± 0.409	1.559 ± 0.122
	CI (F1)	1.585 ± 0.327	1.590 ± 0.129

The predictions from the BNN were qualitatively similar, but slightly underperformed when compared with the DE of neural networks (Table 2). In general, the BNN had a slightly larger prediction error for the subglottal pressure. The linear regression had a slightly lower *R*1 value of 0.712. The MAE was 264 Pa, and the MAPE was 24.4%, both of which are slightly larger than those for the DE (Table 2).

**Fig. 2.**
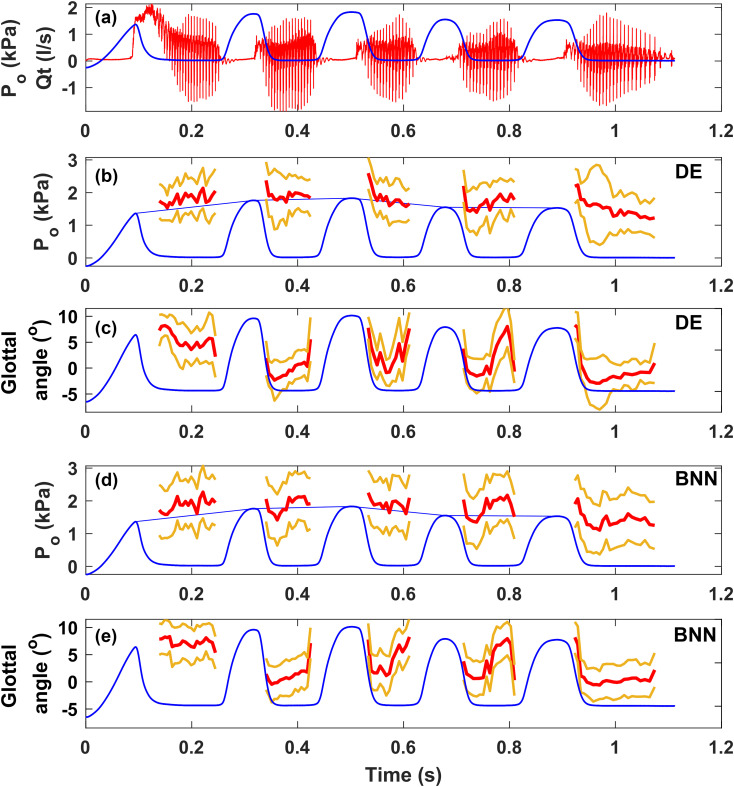
(a) The experimentally measured intraoral pressure (blue) and oral airflow (red), (b) the deep ensemble (DE) estimated subglottal pressure (red) and the 95% CI (yellow) compared with the intraoral pressure, the linear interpolation (blue) of which was used as the ground truth subglottal pressure, (c) the deep ensemble (DE) estimated glottal angle (red) and the 95% CI (yellow), (d) the Bayesian neural network (BNN) estimated subglottal pressure (red) and the 95% CI (yellow), (e) the BNN estimated glottal angle (red) and the 95% CI (yellow).

Figure 2 shows an example of the estimated subglottal pressure and glottal angle over the duration of the utterance /papapapapa/ for Male 2. In the figure, the top panel [Fig. 2(a)] shows the experimentally measured intraoral pressure (blue) and oral airflow (red). Figure 2(b) shows the DE-estimated subglottal pressure (red) and the 95% CI (yellow). Also, shown in Fig. 2(b) in blue is the intraoral pressure, the linear interpolation of which was used as an indirect measure of the subglottal pressure (ground truth) over the duration of the vowels. Comparison between the neural network estimates and the experimental measurement shows that the estimated subglottal pressure generally followed the trend of the experimentally measured subglottal pressure. Except for a few data points [mostly around the transition from /p/ to /a/; not shown in Fig. 2(b)], the experimental values were within the 95% CI of the neural network predictions, with a 99.6% PIC. The mean confident interval (0.528 kPa for all subjects) was narrower than that reported in Ref. 13, which was about 0.88 kPa and higher for human data. While a high prediction interval of coverage suggests reliability and is desirable, it also means that the calculation of the CI may be improved to provide a narrower CI.

Figure 2(c) shows the glottal angle (red) estimated by the DE and its CI (yellow). While experimental measurement of the glottal angle was not available, the estimated glottal angle shows a pattern of alternating adduction (decreasing glottal angle) when the /p/ transitions to /a/ and abduction (increasing glottal angle) when the /a/ transitions to /p/, as would be expected. The general trend of the glottal angle is also consistent with the measured oral airflow waveform. For example, the peak-to-peak amplitude of the oral flow waveform is the largest in the second /pa/ and decreases toward the third and fourth /pa/ before increasing again toward the end of the utterance. This is consistent with the general trend of the minimum glottal angle as predicted by the neural networks. Also, the first /pa/ transition [around 0.15 s in Fig. 2(a)] exhibits a slow phonation onset and high airflow, indicating a gradual and weak adduction. This is consistent with the relatively large glottal angle that decreases slowly during the first /pa/ transition in Fig. 2(c). In contrast, the decrease in the glottal angle is much faster in later /pa/s, which results in almost immediate onset of oral airflow oscillation.

The estimations from the BNN are shown in Fig. 2(d) (subglottal pressure) and Fig. 2(e) (glottal angle). The estimated subglottal pressure also followed the trend of the experiment, although the differences between the estimated subglottal pressure and experimental measurement were slightly larger than those from the DE of neural networks [compare Figs. 2(b) and 2(d)], as also shown in Table 2. The 95% CI predicted by the BNN, however, was much larger than that from the DE of neural networks (Table 2). For example, for Male 2, the 95% confident interval on average was 0.757 kPa for the BNN, compared with 0.6 kPa for the DE of neural network.

For the predicted glottal angle [Figs. 2(c) and 2(e)], the CI was comparable between the two approaches. However, the glottal angle predicted by the BNN [Fig. 2(e)] was generally higher than those predicted by the DE in Fig. 2(c). The BNN also failed to predict the gradual vocal fold adduction at the beginning of the first /pa/ transition [around 0.15 s in Fig. 2(e)], during which the predicted glottal angle stayed more or less the same, whereas a gradual vocal fold adduction was predicted by the DE in Fig. 2(c). While experimental data were not available to quantitatively evaluate the prediction accuracy of the glottal angle, these observations suggest a higher error in the prediction of the glottal angle by the BNN.

### 3.2 Ability to predict meaningful differences across subjects

**Fig. 3.**
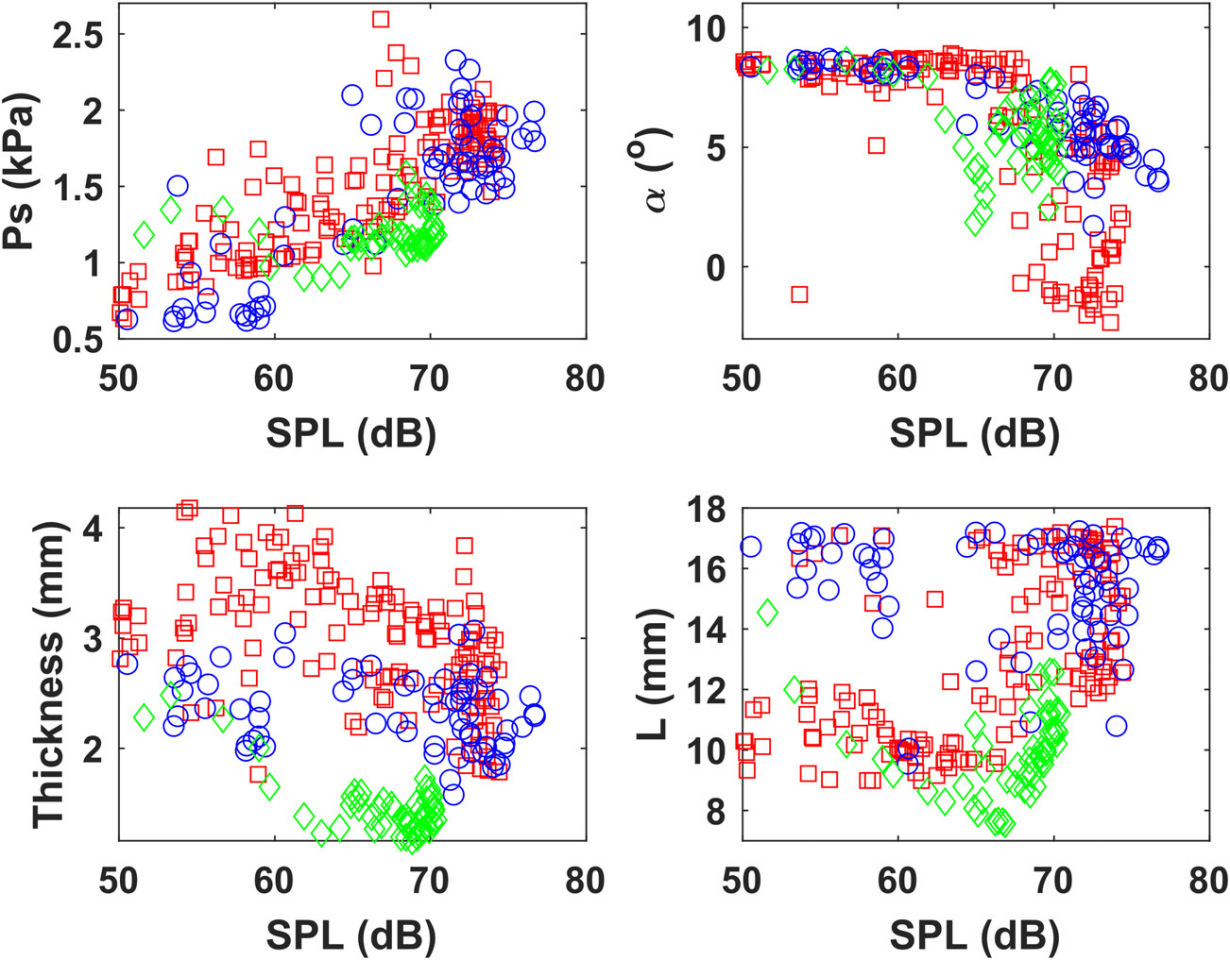
DE estimates of physiological parameters as a function of SPL for all three subjects M1 (blue), M2 (red), and F1 (green).

Figure 3 shows the DE-estimated subglottal pressure, glottal angle, vocal fold vertical thickness, and vocal fold length as a function of the radiated SPL for all three subjects, each coded by a different color. In general, when increasing vocal intensity, all three subjects increased the subglottal pressure and decreased the glottal angle (i.e., increased vocal fold adduction). However, the degree of change was speaker specific. For example, subject Male 2 (red in Fig. 3) tended to approximate his vocal folds more tightly than the other subjects (smaller glottal angle; upper right panel in Fig. 3) and adopted a thick vocal fold configuration (lower left in Fig. 3). This combination of thick vocal folds and tight approximation is known to be associated with high risk of vocal fold injury.^19^ The neural networks were also able to predict meaningful sex-related differences between males and females. Figure 3 shows that the female subject (green) tended to have the thinnest and shortest vocal folds, as would be expected. Although not shown here, similar observations can be made from predictions from the BNN, despite the wider CIs and potentially higher prediction errors.

## 4. Conclusion

The goal of this study was to evaluate the feasibility of two approaches for voice inversion with uncertainty quantification toward monitoring vocal behavior at the physiological level in human subjects. Both the DE and the BNN were able to predict the subglottal pressure with reasonable accuracy, with prediction errors comparable to previous studies.^8–13^ Comparison with experiment shows that the experimental truth was within the calculated CIs, with the predicted CIs narrower than that in a previous study^13^ and with a 99.6% PIC. Both approaches were also able to qualitatively predict the alternating vocal fold adduction/abduction behavior during consonant-vowel transitions. When applied to data from the three human subjects, both approaches were able to identify meaningful differences across subjects in how they modulated the subglottal pressure and vocal fold adduction, two important parameters contributing to vocal health, as well as sex-related differences in vocal fold length and thickness. This indicates that this simulation-based machine learning approach has the potential toward clinical applications such as early detection of unhealthy vocal behavior and ambulatory monitoring of vocal behavior both inside and outside the clinic.

In this study, the DE slightly outperformed the BNN, with the DE having smaller MAE and narrower CIs. Similar observation (deep ensembles outperforming BNNs) was reported in the literature [e.g., Refs. 15 and 17]. It is noted that the goal of this study was not to compare these two methods. No effort was made in this study to optimize the configuration of the BNN. It is very likely that the performance of the BNN can be improved by better approximating the posterior distributions, which is worth pursuing in future studies.

The CI for the subglottal pressure is still relatively large (around 500 Pa for the DE). The high PIC, while desirable, may also indicate inherent variability in the predictions, the sources of which need to be investigated to further narrow the CIs. Many reasons might contribute to the relatively large CIs. For example, the data used to train the neural networks were from computational simulations with each of the model control parameters varying only in a small number of fixed levels (mostly less than four levels except for the subglottal pressure). Thus, the data represent a uniform and coarse sampling of the model parameter space, which may not be ideal for training the neural networks, considering the highly nonlinear nature of the physics of voice production.^23^ In addition, the training data were generated from simulations without a vocal tract, thus neglecting source–filter interaction, and inverse filtering was used to extract voice source information from the human data. While source–filter interaction is expected to be weak in the speech range, it may impact the glottal aerodynamic measures^24,25^ and introduce errors in voice source measures,^26,27^ thus negatively impacting the performance of voice production inversion. Ideally, this issue can be avoided by using simulation data obtained with a vocal tract. However, this will significantly increase the number of model controls and the number of simulations beyond our limited computing resources. These issues will be addressed in future studies.

Only three subjects were included in this study and quantitative validation was limited to the subglottal pressure only. Toward clinical applications, future studies should focus on validation in a large number of subjects performing diverse speech tasks, and validation of other physiological parameters against experimental data, including those from electroglottography or high-speed vocal fold imaging.

## Data Availability

The data that support the findings of this study are available from the author upon reasonable request.
